# Effect of Dietary Patterns on Long‐Term Weight Maintenance of Patients After Sleeve Gastrectomy

**DOI:** 10.1002/fsn3.70834

**Published:** 2025-09-12

**Authors:** Shuwen Zheng, Aihua Li, Yuxian Yang, Dong Zhao, Jing Ke

**Affiliations:** ^1^ Center for Endocrine Metabolism and Immune Diseases, Beijing Luhe Hospital Capital Medical University Beijing China; ^2^ Laboratory for Clinical Medicine Capital Medical University Beijing China

**Keywords:** dietary pattern, obesity, sleeve gastrectomy, weight maintenance

## Abstract

Obesity has emerged as a significant global public health challenge. Sleeve gastrectomy (SG), currently the most widely performed bariatric procedure worldwide, has demonstrated substantial efficacy in achieving significant weight loss and improving metabolic health. However, notable postoperative complications—particularly malnutrition and weight regain—require heightened clinical attention and the implementation of effective long‐term management strategies. These adverse outcomes result from a variety of factors, including anatomical changes that trigger complex hormonal, metabolic, and immune adaptations. These issues are often compounded by suboptimal lifestyle behaviors and poor adherence to postoperative recommendations. Emerging evidence highlights that sustained dietary counseling provides significant long‐term benefits for the majority of patients undergoing SG. Therefore, this review aimed to synthesize the current understanding of the pathophysiological mechanisms that drive digestive adaptations, malnutrition, and weight regain following SG. Further, it evaluates the efficacy, feasibility, and tolerability of targeted dietary management strategies aimed at mitigating these complications.

## Graphical abstract

This review synthesizes current knowledge on the pathophysiology of gastrointestinal adaptations, malnutrition, and weight regain following sleeve gastrectomy. It further evaluates the efficacy, feasibility, and tolerability of targeted dietary management strategies to address these complications.

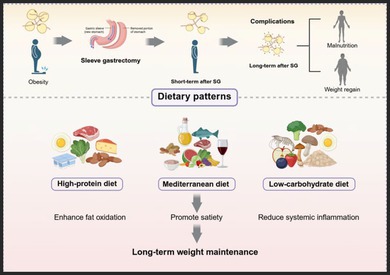

**Abbreviations**
GLP‐1: glucagon‐like peptide‐1
GLP‐2: glucagon‐like peptide‐2
IWL: inadequate weight loss
LC: low‐carbohydrate diet
MedDiet: Mediterranean diet
OXM: oxyntomodulin
PYY: peptide YY
RYGB: Roux‐en‐Y gastric bypass
SG: sleeve gastrectomy
TBARS: thiobarbituric acid reactive substances
VLCKD: very low‐calorie ketogenic
WR: weight regain

## 1 Background

Obesity is defined as the abnormal or excessive accumulation of adipose tissue and has emerged as an international public health issue (Jivraj 2016). There are more than 1.9 billion overweight and approximately 650 million obese adults worldwide (Ruban et al. 2019). Prediction models indicate that the prevalence of adult obesity could reach as high as 42% by the year 2030 (Finkelstein et al. 2012). Obesity is a well‐recognized risk factor for the development of comorbid conditions including cardiovascular diseases, diabetes mellitus, malignancy, obstructive sleep apnea, nonalcoholic fatty liver disease, and gallbladder disorders (Jehan et al. 2020). Bariatric surgery is currently the most effective strategy for treating obesity and type 2 diabetes mellitus (Sandoval and Patti 2023). When conservative treatments fail to achieve satisfactory results in patients with obesity, sleeve gastrectomy (SG) and Roux‐en‐Y gastric bypass (RYGB) are the most commonly performed bariatric procedures (Ali et al. 2017; Angrisani et al. 2018, 2017). For patients with long‐standing, refractory type 2 diabetes mellitus with relatively low serum C‐peptide levels, RYGB is generally recommended, whereas SG is preferred for other patients with morbid obesity (Guraya and Strate 2020; Sandoval and Patti 2023). The effectiveness of these two procedures in achieving weight loss has been investigated in several studies (Table 1; Gronroos et al. 2021; Han et al. 2020; Murphy et al. 2022; Peterli et al. 2013, 2018; Salminen et al. 2022; Shoar and Saber 2017; Zhao and Jiao 2019). However, there are currently no standardized criteria for defining inadequate weight loss (IWL) and weight regain (WR) following bariatric surgery. Additionally, a subset of patients undergoes revision surgery to address WR. Thus, comparisons of IWL and WR between RYGB and SG have not been adequately conducted (Noria et al. 2023; Zhao and Jiao 2019). Interestingly, although RYGB has long been regarded as the gold standard for bariatric surgery, surgeons are increasingly favoring SG due to its shorter operative time and comparable weight loss outcomes (Guraya and Strate 2020). In SG, a sleeve‐like pouch is created that connects the esophagus to the small intestine. Gastric volume reduction directly affects nutrient intake, digestion, and absorption, as well as gut hormone secretion (Steenackers, Van der Schueren, et al. 2018). Anatomical and functional changes in the gastrointestinal tract contribute to both weight loss and associated metabolic improvements, while nutrient deficiencies and WR tend to increase over time (Bal et al. 2012; Steenackers, Gesquiere, and Matthys 2018). The dietary recommendations for patients undergoing SG have been well‐established (Ha et al. 2021). However, these recommendations are often complex and are frequently not followed by patients in practice. In this article, we review the possible mechanisms of weight loss and WR after SG, as well as current dietary strategies.

**TABLE 1:** Comparison of weight loss outcomes between two procedures, RYGB and SG.

Study (author/year)	Study design	Sample size	Crowd characteristics	Follow‐up time	The primary end point	Outcomes
Peterli et al. (2018)	RCT	*N* = 217 LSG (*n* = 107) or LRYGB (*n* = 110)	Mean age, 45.5 years; 72% women; mean BMI, 43.9	5 year	%EWL^a^	No significant difference
Zhao and Jiao (2019)	Meta‐analysis	11 studies (*N* = 1328 participants)	Body mass index (BMI) > 27.5 kg/m^2^, aged > 18 years	Midterm outcomes: 12–36 months; Long‐term outcomes: after 36 months		No significant difference
Shoar and Saber (2017)	Meta‐analysis	14 studies (*N* = 5264 patients)	(BMI) > 27 kg/m^2^ or aged > 18 or < 65 years old	Midterm (3–5 years) and long term (≥ 5 years)		No significant difference between LRYGB and LSG in midterm weight loss but revealed a significant difference in long‐term weight loss
Salminen et al. (2022)	RCT	*N* = 240 patients (121 = LSG and 119 = LRYGB)	167 women [69.6%]; mean age = 48.4 years; mean baseline BMI = 45.9	10 years	%EWL^a^	No significant difference
Han et al. (2020)	Meta‐analysis	20 studies (*N* = 2917 participants)	(BMI) ≥ 40 kg/m^2^ or ≥ 35 kg/m^2^ with one or more comorbid conditions; aged of 18–60 years	Midterm outcomes: 12–36 months; Long‐term outcomes: after 36 months		No significant difference
Gronroos et al. (2021)	RCT	240 patients LSG (*n* = 121) or LRYGB (*n* = 119)	167 women [69.6%]; mean age = 48.4 years; mean baseline body mass index = 45.9	7 years	%EWL^a^	No significant difference
Murphy et al. (2022)	RCT	114 adults LRYGB = 57 or LSG = 57	Type 2 diabetes and BMI 35–65 kg/m^2^	5 years	%AWL^b^ or%EWL^a^	LRYGB provided superior weight loss compared with LSG
Peterli et al. (2013)	A Prospective Randomized Trial	*N* = 217 LSG = 117 LRYGB = 110	BMI = 44 ± 11.1 kg/m^2^, the mean age = 43 ± 5.3 years, 72% female	1 year	Excessive BMI loss	No significant difference

Abbreviations: LRYGB, laparoscopic Roux‐en‐Y gastric bypass; LSG, laparoscopic sleeve gastrectomy.

^a^ Percentage excess weight loss (%EWL) is calculated as (initial weight − follow‐up weight)/(initial weight − ideal weight for BMI 25) × 100.

^b^ Percent absolute weight loss (%AWL) is calculated as ([baseline weight − follow‐up weight]/[baseline weight]) × 100.

## 2 The Impact of SG on Diet, Digestion, and Absorption

Following SG, patients' dietary habits and tolerances undergo significant changes. Carbohydrates such as noodles are generally well‐tolerated (Diaz‐Lara et al. 2020; Kvehaugen and Farup 2018; Ruiz‐Tovar et al. 2018). Among meats, chicken, turkey, rabbit, minced meats, and all types of fish are usually well‐tolerated (Diaz‐Lara et al. 2020; Ramon et al. 2012). Vegetables such as mushrooms, pumpkin, zucchini, chard, green beans, and spinach—typically cooked at high temperatures—are also well‐tolerated (Diaz‐Lara et al. 2020). A prospective study found that yogurts, skim milk, and cottage cheese were well‐tolerated, whereas full‐fat milk was poorly tolerated (Coluzzi et al. 2016). Most liquids are well‐tolerated, with broth, infusions, and juices being the most well‐tolerated. Wine is poorly tolerated, while carbonated beverages—generally not recommended—show the lowest tolerance (Coluzzi et al. 2016). Although patients following SG may experience intolerance to certain foods, studies indicate this intolerance is often temporary and tends to improve over time (Ruiz‐Tovar et al. 2018). Regarding food preferences, numerous studies report a decreased desire for sweets, fats, salty foods, and alcohol, along with an increased preference for tart foods following SG (Coluzzi et al. 2016; Nance et al. 2020; Schiavo et al. 2022). However, some studies have found no significant changes in food preferences after SG (Alabduljabbar et al. 2023; Corbeels et al. 2020; Nielsen et al. 2017; Sondergaard Nielsen et al. 2018).

In addition to altering food intake and preferences, SG also affects nutrient digestion and absorption (Janmohammadi et al. 2019). Partial resection of the stomach affects gastric digestion and reduces the secretion of gastric acid and other intrinsic factors (Bal et al. 2012). As a result, reduced gastric mixing and accelerated gastric emptying facilitate rapid transport of some of the undigested nutrients to the small intestine. In this situation, proteins, carbohydrates, and fats may be digested more slowly. Impaired lipid digestion can lead to malabsorption of fat‐soluble vitamins (A, D, E, and K; Steenackers et al. 2021; Vinolas et al. 2019).

**FIGURE 1:**
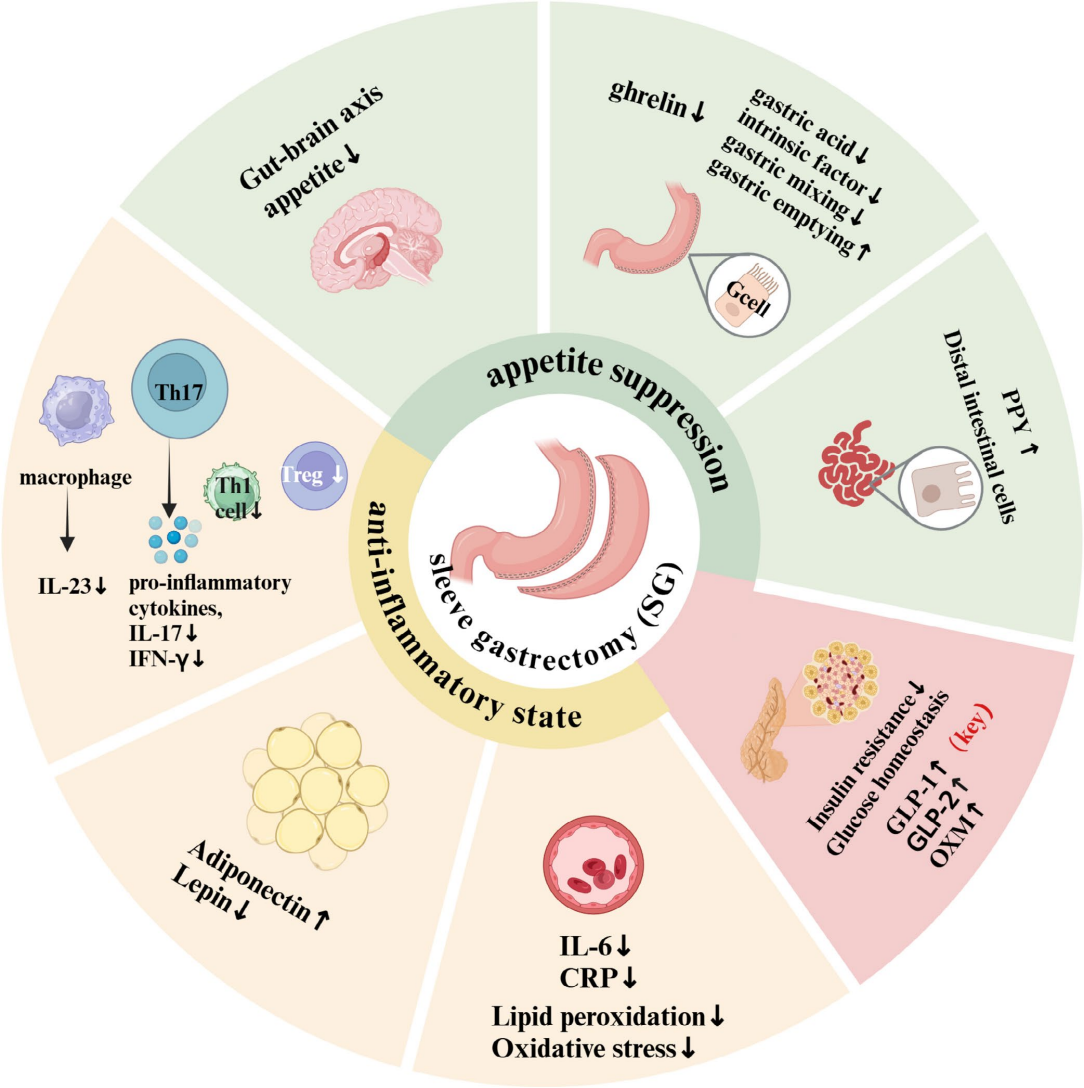
The mechanism of weight loss after SG surgery. SG removes most of the stomach, reducing ghrelin‐producing cells. Faster gastric emptying post‐SG boosts PYY secretion from distal enterocytes, which slows intestinal transit and enhances satiety. Reduced gastric acid and increased pH also promote PYY release. GLP‐1 rises after meals, further suppressing appetite and enhancing satiety. SG decreases leptin due to reduced fat mass and nutrient absorption, affecting energy regulation. It also improves insulin secretion and sensitivity. Together, these hormonal shifts support appetite suppression, weight loss, glucose control, and reduced inflammation. CRP, C‐reactive protein; GLP‐1, glucagon‐like peptide‐1; GLP‐2, glucagon‐like peptide‐2; OXM, oxyntomodulin; PYY, peptide YY; (Created with Biorender).

Bariatric surgery impacts nutrient absorption primarily through neurohumoral mechanisms involving the gut–brain axis. Postoperative increases in gut hormones such as glucagon‐like peptide‐1 (GLP‐1), GLP‐2, oxyntomodulin (OXM) and peptide YY (PYY) are commonly observed (Fedonidis et al. 2014; McCarty et al. 2020), with GLP‐1 and PYY playing key roles in glucose homeostasis after surgery (Gu et al. 2021). Studies also reported reduced oxidative stress markers such as plasma thiobarbituric acid reactive substances (TBARS) (Prior et al. 2017) and urinary 8‐oxo‐dG following SG, indicating improved metabolic status (Monzo‐Beltran et al. 2017). SG modulates glucose and lipid metabolism, contributing to weight loss and the amelioration of low‐grade systemic inflammation, as evidenced by reduced pro‐inflammatory cytokines including IL‐17, IL‐23, and IFN‐γ (Mallipedhi et al. 2014). Our study summarizes changes in dietary intake, digestion, nutrient absorption, gut physiology, hormone levels, and inflammation following SG (Figure 1).

## 3 Malnutrition and WR After SG

### 3.1 Malnutrition After SG

Although individuals with obesity consume excessive calories, they frequently present with nutritional deficiencies and malnutrition prior to surgery. This is likely due to a lower intake of protein‐, vitamin‐, mineral‐, and fiber‐rich foods, combined with excessive consumption of high‐calorie, nutrient‐poor diets (Elhag and El Ansari 2022). Vitamin and micronutrient deficiencies are common after SG (Nie et al. 2023) and can lead to serious complications, including anemia (Steenackers et al. 2023; Zhang et al. 2021), metabolic bone diseases (e.g., osteoporosis, fractures; Aaseth and Alexander 2023; Gibbs 2022), and neurological disorders such as Wernicke's encephalopathy, optic neuropathy, myelopathy, and peripheral neuropathy (Goodman 2015; Hamilton et al. 2018; Schimpke and Guerron 2018). Contributing factors include reduced dietary intake, altered food preferences (Steenackers et al. 2023), postoperative symptoms (e.g., vomiting, nausea, and food intolerance) and poor adherence to dietary guidelines and follow‐up recommendations (Mulita et al. 2021; Zarshenas et al. 2020). Despite these risks, nutritional management is often overlooked. A recent meta‐analysis reported that adherence to supplementation guidelines remains below 20% post‐SG (Ha et al. 2021), with forgetfulness, cost, and side effects being the main barriers (Steenackers et al. 2023). Therefore, exploring more affordable and sustainable dietary strategies for SG patients is essential.

### 3.2 WR After SG

WR after SG is an increasing concern (Cohen and Petry 2023). Due to the lack of standardized definitions, the reporting rate of WR varies widely, from 15.4% to 50% (Cohen and Petry 2023; Baig et al. 2019; Ben‐Porat et al. 2021; Clapp et al. 2018; Lopes et al. 2022). A great number of studies have explored both pre‐ and post‐operative risk factors. Dietary behaviors play a central role, with frequent intake of sweetened beverages, high carbohydrates, low protein, and poor adherence to dietary guidelines being strongly linked to WR (Ben‐Porat et al. 2021; Kaouk et al. 2019; Moslehi et al. 2023). Maladaptive eating patterns—including grazing, binge eating, loss‐of‐control eating, and nocturnal eating—are also strongly associated with WR (Ben‐Porat et al. 2022; Cohen and Petry 2023; Noria et al. 2023; Palacio et al. 2021). Beyond behavior, higher preoperative BMI (Body Mass Index) and younger age are notable predictors of WR (Ben‐Porat et al. 2021), although findings on age remain inconsistent. Some studies associate WR with older age (> 60 years; Al‐Khyatt et al. 2017; Bakr et al. 2019; Paul et al. 2017) while others implicate younger patients (Shantavasinkul et al. 2016). Additionally, older age is also linked to IWL at 1 year post surgery (Cadena‐Obando et al. 2020). Sex differences are more consistently observed in studies related to WR and other postoperative outcomes (Ma et al. 2006; Melton et al. 2008; Mocanu et al. 2020). Males tend to experience poorer outcomes—including suboptimal weight loss (Ma et al. 2006) and greater risk of comorbidity recurrence—whereas females typically achieve greater weight loss and are more resistant to WR (Stroh et al. 2014; Mocanu et al. 2020; Stroh et al. 2014). This disparity may stem from sex‐specific metabolic factors such as central adiposity and insulin resistance in males versus estrogen‐related insulin sensitivity and higher adiponectin levels in females (Masharani et al. 2009; Geer and Shen 2009). Furthermore, anatomical changes such as gastric band slippage, fistula enlargement, fundal dilatation, or gastro‐jejunostomy can also contribute to WR. In addition, binge eating associated with depression or emotional distress confers elevated WR risk (Bakr et al. 2019; Cohen and Petry 2023; Noria et al. 2023; Palacio et al. 2021; Pizato et al. 2017; Romagna et al. 2023).

### 3.3 Mechanisms Underlying WR Following Bariatric Surgery

**FIGURE 2:**
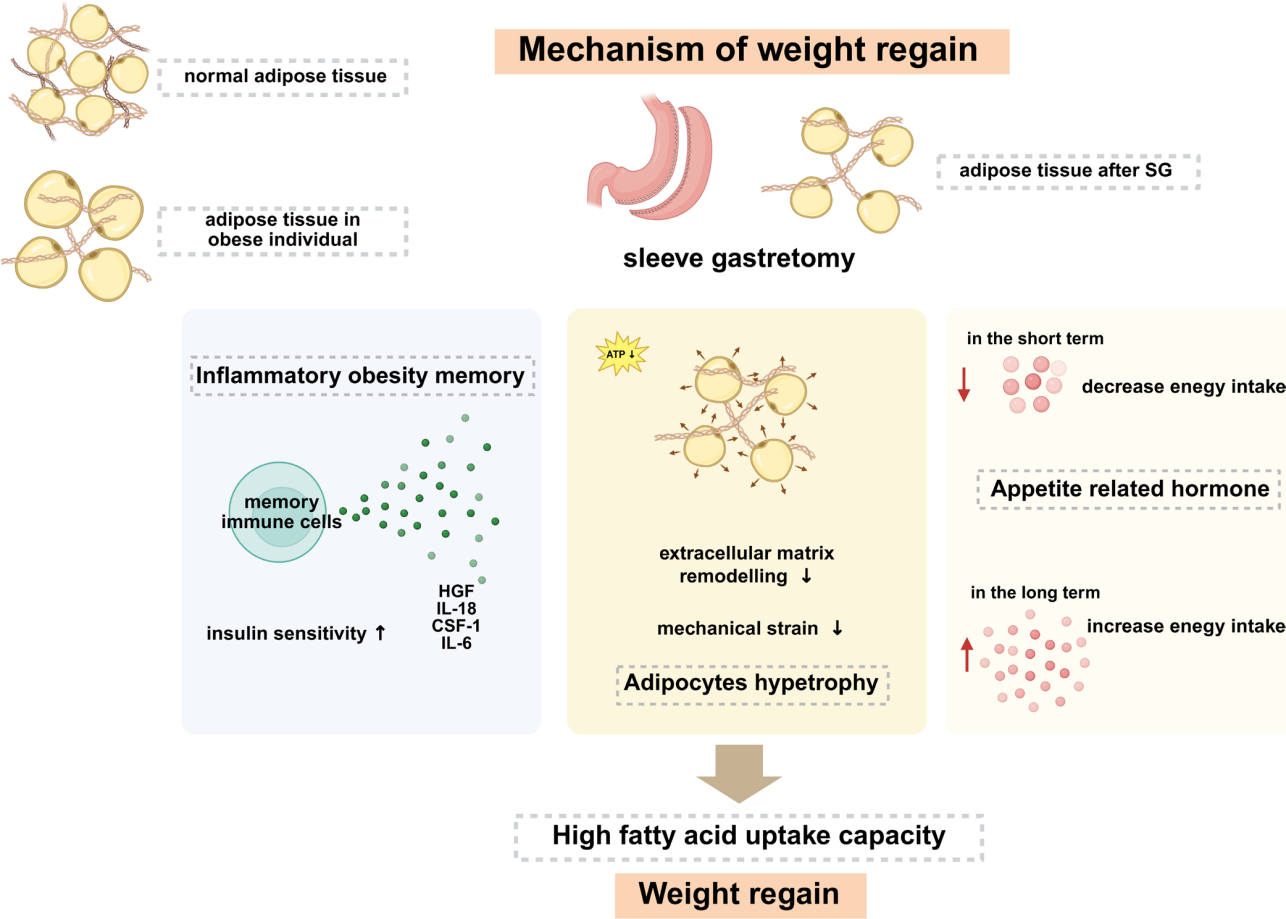
The mechanism of weight regain after SG surgery. Following SG, growth hormone levels acutely decline but frequently normalize over time. In an energy‐deficient state, calorie restriction impairs extracellular matrix remodeling required for adipocyte contraction. It increases mechanical stress and fat re‐accumulation. Adipocytes retain high fatty acid uptake capacity despite volume reduction. Additionally, “inflammatory obesity memory” persists in immune cells, while specific metabolic markers and innate immune mediators (e.g., HGF, IL‐18, CSF‐1) are associated with WR. In this figure, the purple, yellow, and pale yellow sections represent key factors involved in WR after SG: The immune system, extracellular matrix components, and appetite‐related hormones. SG, sleeve gastrectomy; WR, weight regain; (Created with BioRender).

Emerging evidence highlights the interconnected roles of inflammation, metabolic activation, and innate immunity in WR following dietary and pharmacologic interventions (Figure 2), although their relevance to WR after SG remains unclear. Hormonal changes post‐SG likely mediate WR risk. Ghrelin levels decrease acutely after gastric bypass but often return to baseline over time, potentially increasing energy intake and contributing to WR (Dimitriadis et al. 2017; Sherf‐Dagan et al. 2019). Other appetite‐regulating hormones such as GLP‐1, PYY, leptin, and cholecystokinin have also been linked to post‐surgical WR (Sherf‐Dagan et al. 2019).

Adipose tissue dynamics further influence WR. During energy deficit, adipocyte contraction requires extracellular matrix remodeling. However, caloric restriction may impair this process, leading to mechanical strain that inhibits lipolysis and promotes fat re‐accumulation (van Baak and Mariman 2019). Adipocytes in obese individuals preferentially uptake fatty acids (Schwartz et al. 2017), and post‐bariatric adipocytes retain high uptake capacity despite reduced size (Ge et al. 2016), and enhancing storage efficiency compensates for reduced fat mass, potentially facilitating SG‐related regain. Elevated fat mass at 6–12 months post surgery may predict metabolic risk and WR (Sherf‐Dagan et al. 2019).

Immune‐metabolic memory also plays a role in WR after sugery. An “inflammatory obesity memory” can persist in immune cells even after weight loss induced by medication (van Baak and Mariman 2023). Additionally, certain metabolic markers such as fasting insulin, IL‐6, leukocytes, adipose macrophages have been associated with an increased risk of WR (Kong et al. 2013). Innate immune mediators such as HGF, IL‐18, CSF‐1, regulate appetite, insulin sensitivity and adipose tissue function in obesity models (Perry et al. 2023), underscoring the importance of investigating immune activation in post‐surgical WR. Despite growing evidence, the pathophysiological mechanisms driving WR remains incompletely understood, highlighting the need for further research to support personalized management strategies.

### 3.4 Therapeutic Strategies for WR

Interventions for WR mainly include dietary changes, behavior therapy, medication, and revisional surgery. Targeting unhealthy eating habits and physical inactivity may help prevent WR (Herring et al. 2017; Himes et al. 2015). The effectiveness of behavioral and lifestyle intervention for WR has been partially validated (Bradley et al. 2016, 2017; Horber and Steffen 2021; Noria et al. 2023; Wharton et al. 2019). Anti‐obesity medications seem to be less effective after SG, and more randomized controlled trials are needed to assess the benefit of combining pharmacotherapy with lifestyle changes (Noria et al. 2023). Revisional surgery, particularly, RYGB is the most commonly used approach for reversing WR after SG and may be effective in some cases, though it carries higher complication risks (Noria et al. 2023). Endoscopic options are emerging alternatives (Cohen and Petry 2023; Baig et al. 2019; Cohen and Petry 2023). Overall, current evidence suggests limited success with behavioral, exercise, and pharmacological interventions post‐SG, highlighting the urgent need for robust RCTs on integrated treatment strategies.

## 4 Effects of Different Dietary Patterns on Weight Loss and Weight Maintenance After SG

While SG is effective for obesity, long‐term success depends on proper dietary management to prevent WR, malnutrition, and other complications. In the short term (6–12 months post‐SG), the focus is on adequate protein and fluid intake to preserve muscle mass. Long‐term management (> 1 year) emphasizes controlling energy intake and supplementing nutrients to prevent WR and deficiencies. Consistent intake of protein, vitamins, and minerals is essential throughout. Overall, tailored dietary strategies are crucial at each stage to sustain weight loss and support metabolic health.

Postoperative behavioral management may enhance weight loss and long‐term maintenance. A meta‐analysis of 13 studies found greater weight loss with behavioral management versus usual care or no treatment (Rudolph and Hilbert 2013). Current bariatric guidelines emphasize dietary recommendations for the first postoperative year, yet evidence on long‐term diet quality and optimal composition remains limited (Allied Health Sciences Section Ad Hoc Nutrition et al. 2008; Kanerva et al. 2017; Moize et al. 2010). High‐protein, Mediterranean, and low‐carbohydrate diets show promise for sustaining long‐term weight loss in SG patients (Table 2). However, establishing evidence‐based dietary guidelines requires systematic evaluation of the current literature.

**TABLE 2:** Comparison of different dietary patterns outcomes among patients since SG.

Study (author/year)	Study design	Characteristics of the participants	Dietary pattern	Follow‐up time	The primary end point	Outcomes
Moslehi et al. (2024)	Cross‐sectional study	*N* = 146; age (43.6 ± 12.1;77.4% females)	The dietary pattern 1 is characterized by high intakes of fast foods, sauce, soft drinks, processed meats, sugar confectionery, salty snacks, grains, organ meats, poultry and fish, animal fat, and vegetable oil. The dietary pattern 2 is featured with high consumption of fruits, dairy, vegetables, legumes, eggs, nuts, red meats, and poultry and fish	2–4 years	%FML^b^ > 77.9%; %FFML^c^ > 28%; %TWL^a^ < 25%	Dietary pattern 1 has significantly lower %TWL.^a^ lower%FML^b^, higher%FFML^c^, and higher odds of excessive FFM^d^ loss
Lim et al. (2020)	Retrospective observational study	*N* = 43	High‐protein and low‐carbohydrate diet; Low‐protein and high‐carbohydrate diet	1, 3, 6, and 12 months	%EWL^e^ > 50%	The cutoff intakes are > 44.5, > 41.5, and > 86.5 g/day at 1, 6, and 12 months post operation with regard to protein relate to long‐term weight loss
Schiavo et al. (2020)	Prospective cohort study	*N* = 74 (78.4% females, age 43 ± 8.2)	The Mediterranean diet	4 years	%EWL^e^ > 50%	Weight regain appears in 37.8% of participants followed by the IMD recommendations
Sherf Dagan et al. (2017)	Prospective cohort study	*N* = 68; age (42.7 ± 9.4)	High‐protein diet	6 and 12 months	FFM^d^ loss with a cutoff of 10%	Protein intake of ≥ 60 g/day is associated with a significantly lower relative FFM^d^ loss
Chou et al. (2017)	Retrospective study	*N* = 40	High‐protein diet and low‐carbohydrate diet	5 years	%TWL^a^ < 25%	Higher protein intake is associated with reduced weight regain

^a^ %TWL = preoperative weight − postoperative weight/preoperative weight × 100.

^b^ %FML = (preoperative FM − postoperative FM)/preoperative weight − postoperative weight × 100.

^c^ %FFML = (preoperative FFM − postoperative FFM)/preoperative weight − postoperative weight × 100.

^d^ FFM = Body weight − (Body weight × Fat%).

^e^ %EWL = (weight loss/excess weight × 100, where excess weight = total weight before prebariatric surgery 2Ȓ ideal weight).

### 4.1 High‐Protein Diet

To prevent short‐term WR after SG, current dietary guidelines recommend a high‐protein (≥ 35% of energy), low‐carbohydrate (≤ 45%) and low‐fat (≤ 20%) diet (Mechanick et al. 2013; Moize et al. 2010). A protein intake of 60–80 g/day or 1.2–1.5 g/kg of ideal body weight helps preserve muscle mass and basal metabolic rate (Mechanick et al. 2019). Studies show a strong link between high protein intake and greater weight loss post‐bariatric surgery (de Souza Vilela et al. 2023). A 10‐year prospective longitudinal study of 1610 Swedish patients revealed that those with higher protein and lower fat intake maintained greater weight loss (17.2% and 20.3% total weight loss, respectively; Kanerva et al. 2017). Protein also supports wound healing and facilitates the transition to an oral diet. Protein promotes satiety more effectively than carbohydrates or fats by stimulating anorexigenic hormones, raising plasma amino acids, increasing thermogenesis, and promoting gluconeogenesis, which together enhance fat oxidation (de Souza Vilela et al. 2023; Romeijn et al. 2021). Given SG‐induced hormonal and metabolic changes, adherence to a high‐protein diet may help sustain weight loss and reduce WR risk after SG.

### 4.2 The Mediterranean Diet (MedDiet)

MedDiet, rich in plant‐based foods and olive oil with moderate animal product intake, demonstrates strong potential for sustaining weight loss after SG. In a study with over four years of follow‐up, 37.8% of SG patients experienced WR, primarily due to poor adherence to the MedDiet, highlighting its importance for long‐term success in weight maintenance (Schiavo et al. 2020). Mechanistically, the MedDiet supports weight maintenance by improving lipid profiles, reducing inflammation, modulating mTOR via amino acid restriction, and promoting beneficial microbiota metabolites (Tosti et al. 2018).

Key MedDiet components‐vegetables, fruits, olive oil, nuts, and wine‐offer antioxidants and anti‐inflammatory compounds that support post‐bariatric weight loss and reducing comorbidities. These effects are associate with slower cellular aging (Canudas et al. 2020) and lower inflammation, especially with extra‐virgin olive oil (Calder 2022). Though low in saturated fat (< 8% of calories), the MedDiet is high in healthy fats (25%–35%) from omega‐3‐rich sources such as olive oil, nuts and seeds, which improve adipocyte function (Tosti et al. 2018; van Baak and Mariman 2019) and reduce systemic inflammation (Kalupahana et al. 2020). Additionally, its high fiber content enhances satiety by stimulating GLP‐1 and PYY, which slows gastric emptying (Cani and Delzenne 2009). Despite SG reducing stomach volume by 85% and accelerating emptying‐blunting natural satiety signals‐the MedDiet counters these effects by restoring satiety, lowering inflammation and supporting metabolic changes from surgery. This integrated approach provides a sustainable alternative to complexdiets, improving adherence and long‐term outcomes.

### 4.3 Low‐Carbohydrate Diet (LC) and Very Low‐Calorie Ketogenic (VLCKD) Diet

LC reduce carbohydrates intake to promote fat metabolism (Ajala et al. 2013). The American Diabetes Association defines LC as less than 130 g/day of carbohydrates or less than 26% of total energy. The VLCKD diet program typically provides 500–800 kcal/day with < 50 g carbohydrates, 1.2–1.5 g/kg protein (ideal body weight) and moderate fat (15–30 g), inducing ketogenesis. Ketone bodies help regulate appetite. In a study of 22 nondiabetic, obese adults, VLCKD led to a significant weight loss (mean 14%) while preserving muscle strength (Vinciguerra et al. 2023), suggesting potential benefits post‐bariatric “poor responders.” Sufficient protein intake in VLCKD supports gluconeogenesis and prevents muscle loss—a key factor in preventing WR, as muscle loss reduces resting energy expenditure (Volek et al. 2024). Although bariatric surgery is effective for weight loss, research on long‐term dietary management strategies remains limited. Current focus is on the MedDiet and high‐protein diets, though their impact on body composition and nutrition needs further study.

## 5 Conclusions

Obesity contributes to lifestyle‐related diseases such as diabetes, fatty liver, and cardiovascular disorders. Bariatric surgery, particularly SG, is a common intervention. SG alters digestion and nutrient absorption by reducing gastric acid, enzymes, mixing, and increasing emptying. It also changes food tolerance and preferences. While SG achieves effective weight reduction, persistent postoperative concerns require attention. Due to poor adherence to supplementation and dietary advice, malnutrition, and WR are common after SG. Therefore, this review focuses on identifying effective and accessible dietary alternatives. Dietary patterns such as high‐protein, MedDiet, and LC diets may support weight maintenance; however, further research is needed to clarify their effectiveness and mechanisms following SG. Prospective studies are essential to identify which patients benefit most from specific dietary strategies, thereby informing long‐term management. Physicians should assess preoperative nutritional status and eating habits based on individual needs, food preferences, activity levels, and daily routines. Proactive nutritional supplementation before surgery, combined with vigilant postoperative management and regular follow‐up, can enhance intervention success and prevent nutritional deficiencies. In summary, routine follow‐up, personalized nutrition strategies, and timely correction of nutrient deficiencies are key to optimizing SG outcomes and reducing related public health burdens.

## Author Contributions

**Dong Zhao:** conceptualization (equal); writing – review and editing (equal). **Jing Ke:** conceptualization (equal); writing – review and editing (equal). **Shuwen Zheng:** writing – original draft (lead); visualization (equal). **Aihua Li:** writing – original draft (equal); visualization (equal). **Yuxian Yang:** writing – original draft (equal); visualization (equal).

## Ethics Statement

The authors have nothing to report.

## Consent

The authors have nothing to report.

## Conflicts of Interest

The authors declare no conflicts of interest.

## Data Availability

The authors have nothing to report.
